# Histologic Patterns and Clues to Autoinflammatory Diseases in Children: What a Cutaneous Biopsy Can Tell Us

**DOI:** 10.3390/dermatopathology8020026

**Published:** 2021-06-08

**Authors:** Athanassios Kolivras, Isabelle Meiers, Ursula Sass, Curtis T. Thompson

**Affiliations:** 1 Department of Dermatology and Dermatopathology, Saint-Pierre, Brugmann and Queen Fabiola Children’s University Hospitals, Université Libre de Bruxelles, 1000 Brussels, Belgium; isabelle.meiers@stpierre-bru.be (I.M.); ursula.sass@stpierre-bru.be (U.S.); 2 Department of Pathology, Laboratoire Luc Olivier, 5380 Fernelmont, Belgium; 3 Department of Dermatology, Oregon Health and Sciences University, Portland, OR 97239, USA; curtisinportland@gmail.com; 4 Department of Pathology, Oregon Health and Sciences University, Portland, OR 97239, USA; 5 CTA Pathology, Portland, OR 97223, USA

**Keywords:** autoinflammation, cryopyrin, inflammasome, interferonopathies, pustular psoriasis, lupus erythematosus, neutrophilic urticarial dermatitis, pyoderma gangrenosum, suppurative hidradenitis

## Abstract

Autoinflammation is defined by aberrant, antigen-independent activation of the innate immune signaling pathways. This leads to increased, pro-inflammatory cytokine expression and subsequent inflammation. In contrast, autoimmune and allergic diseases are antigen-directed immune responses from activation of the adaptive immune system. The innate and adaptive immune signaling pathways are closely interconnected. The group of ‘complex multigenic diseases’ are a result of mutual dysregulation of both the autoinflammatory and autoimmune physiologic components. In contrast, monogenic autoinflammatory syndromes (MAIS) result from single mutations and are exclusively autoinflammatory in their pathogenesis. Studying the clinical and histopathological findings for the various MAIS explains the phenotypical correlates of their specific mutations. This review aims to group the histopathologic clues for autoinflammation into three recognizable patterns. The presence of these histologic patterns in a pediatric patient with recurrent fevers and systemic inflammation should raise suspicion of an autoinflammatory component in MAIS, or, more frequently, in a complex multigenic disease. The three major histopathological patterns seen in autoinflammation are as follows: (i) the ‘neutrophilic’ pattern, seen in urticarial neutrophilic dermatosis, pustular psoriasis, aseptic neutrophilic folliculitis, and Sweet’s syndrome; (ii) the ‘vasculitic’ pattern seen in small vessel-vasculitis (including hypersensitivity/leukocytoclastic vasculitis, thrombosing microangiopathy and lymphocytic vasculitis), and intermediate-sized vessel vasculitis, mimicking polyarteritis nodosa; and (iii) the ‘granulomatous’ pattern. Beyond these three patterns, there are additional histopathologic clues, which are detailed below. It is important for a dermatopathologist to recognize the patterns of autoinflammation, so that a diagnosis of MAIS or complex multigenic diseases may be obtained. Finally, careful histopathologic analyses could contribute to a better understanding of the various clinical manifestations of autoinflammation.

## 1. Introduction

### 1.1. Innate versus Adaptive Immunity: Autoinflammatory Versus Autoimmune Disease—What the Dermatopathologist Needs to Know

Innate immunity is our first line of defense and rapidly recognizes molecules unique to pathogens via pattern recognition receptors, such as the toll-like receptors. However, there is no memory after repeated exposure. Innate immunity employs macrophages, neutrophils and mast cells, and at a molecular level, complement and antimicrobial peptides. In contrast, adaptive immunity is a second line of defense, utilizing T- and B-lymphocytes. Adaptive immunity is highly specific since highly diverse lymphocytes can target specific antigens. Adaptive immunity remembers these specific antigens upon subsequent exposure to the antigen, with the ability to be reactivated. Adaptive immunity is also self-tolerant as it distinguishes self-antigens from foreign antigens [1].

Dysfunction of innate immunity is rare and results from an antigen-independent hyperactivation of molecular pro-inflammatory pathways, leading to ‘autoinflammatory’ diseases. Dysfunction of adaptive immunity is frequent and results from non-pathogen activation of inflammation. The non-pathogen is a self-antigen in autoimmune diseases and an environmental antigen in allergic diseases. The inherited diseases that cause autoinflammation, called ‘monogenic autoinflammatory syndromes’ (MAIS), are characterized by recurrent fever, increased cytokine expression, and episodic inflammation, resulting in potential end-organ damage. In contrast to autoimmune diseases, the increased, chronically active cytokine expression does not require auto-reactive lymphocytes or immunoglobulins to self-antigens. Although MAIS may be triggered by an infectious pathogen, their hallmark is the persistence of inflammation in the absence of a recognizable infection [2,3,4,5,6].

Despite these differences, autoinflammation and autoimmunity are interlinked. Complex multigenic diseases have different amounts of both autoinflammatory and autoimmune components within their pathogenesis. Complex multigenic diseases include common diseases such as lupus erythematosus (LE), Crohn’s disease, spondyloarthropathies, and type-1 diabetes mellitus, among others. In contrast to complex multigenic diseases, the rarely-encountered group of monogenic syndromes are either entirely autoimmune or autoinflammatory [7].

The MAIS encountered in pediatric dermatology may arise due to four major modes of activation of the innate immunity as follows: (1) interleukin (IL)-1 activation; (2) type-I-interferon (IFN) activation; (3) nuclear-factor-kappa B (NF-κB) activation; and (4) M1 macrophage activation: [1,5,6,8,9,10].

1. IL-1 activation. The binding of an antigen to a pattern recognition receptor activates a pro-inflammatory cascade of intracellular, multimeric protein complexes called ‘inflammasomes.’ Inflammasomes are defined by their sensor proteins, which oligomerize to activate caspase-1, also called IL-1–converting enzyme, leading to proteolytic activation of IL-1b. Multiple inflammasomes are well-understood, including pyrin and cryopyrin. Cryopyrin is also called NLR family pyrin domain containing 3 (NLRP3). MAIS resulting from mutations within inflammasomes are also called ‘inflammasomopathies’. Unleashed IL1-induced inflammation can also result from deficiency of IL1 and IL36 receptor antagonists. Phospholipase C gamma 2 (PLCƔ2) is a cytoplasmic signaling enzyme, which, when recruited to the membrane upon receptor activation, induces the release of endoplasmic reticulum calcium stores, thereby leading to increased intracellular calcium levels and activation of the NLRP3 inflammasome.
2. Type I interferon (IFN) activation (Type I interferonopathies). Autoinflammatory diseases related to IFN activation, also called interferonopathies, reflect aberrant activation of type I IFN pathways (IFN-α and IFN-β), which are involved in antiviral defense. Type I IFN production is triggered by viral RNA or DNA, and interferonopathies may arise through disorders of intracytoplasmic accumulation of endogenous nucleic acid due to their decreased degradation, through inherent, increased intracytoplasmic nucleic acid sensing or through a proteasome dysfunction.
3. NF-κB activation (NFkBopathies). The NF-κB complex is a central signaling hub within the cytoplasm, integrating signals from multiple cell-surface receptors, including TNF receptors and intracellular pattern recognition receptors, like the nucleotide-binding oligomerization domain 2 (NOD2) receptor. NF-κB allows the freeing of several transcription factors, which move to the nucleus and trigger expression of proinflammatory genes. Activation of caspase-activating recruitment domain, member 14 (CARD14) also leads to enhanced NF-κB activity. A20 is a negative regulator of NF-κB and A20 insufficiency also results in an NFkBopathy.
4. M1 macrophage activation. Adenosine deaminase 2 deficiency results in increased pro-inflammatory M1 macrophages (as opposed to anti-inflammatory M2 macrophages).

**Figure 1:**
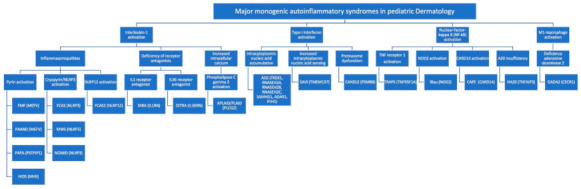
The major monogenic autoinflammatory syndromes in pediatric dermatology based on the major pathogenesis pathways. Mutated genes in parenthesis next to their corresponding syndrome; AGS, Aicardi Goutières Syndrome; APLAID, autoinflammation and phospholipase C gamma 2 associated antibody deficiency and immune dysregulation; CANDLE, Chronic atypical neutrophilic dermatosis with lipodystrophy and elevated temperature; CAPE, Caspase-activating recruitment domain, member 14 associated papulosquamous eruption; DADA2, Deficiency of adenosine deaminase 2; DIRA, Deficiency of IL-1 receptor antagonist syndrome; DITRA, Deficiency of IL-36 receptor antagonist; FCAS, Familial cold autoinflammatory syndrome; FMF, Familial Mediterranean fever syndrome; HA20, Haploinsufficiency of A20; HIDS, Hyperimmunoglobulinemia D syndrome; MWS, Muckle–Wells syndrome; NOMID, Neonatal-onset multisystem inflammatory disease; PAAND, Pyrin-associated autoinflammation with neutrophilic dermatosis; PAPA, Pyogenic Arthritis, pyoderma gangrenosum and acne; PLAID, Phospholipase C gamma 2 associated antibody deficiency and immune dysregulation; SAVI, Stimulator of interferon genes (STING) associated vasculopathy of infancy; TRAPS, TNF receptor-associated periodic syndrome.

Figure 1 summarizes the major activated pathways with their corresponding MAIS and mutated genes. Dysregulation of innate immunity is far more complex than this illustration, which has been simplified for didactic purposes. Of note, this dysregulation of innate immunity is interdependent with adaptive immunity.

### 1.2. Histopathological Clues to the Diagnosis of Autoinflammation

Autoinflammation is devoid of any specific histopathological clue. In fact, different mutations in the same molecular pathway can produce different clinical and histopathological findings. MAIS, which result from single mutations in a pathway, demonstrate close, phenotypic correlations with specific mutations. This information has led to a better understanding of the aberrant activation of the same pathways in the group of complex multigenic diseases [9]. With this knowledge, the dermatopathologist can better recognize histologic patterns associated with autoinflammation and integrate them into a patient’s particular clinical presentation. Beyond recognition, this understanding also provides an opportunity for the use of more targeted treatments. Finally, the identification of histologic patterns associated with MAIS allows for a better understanding and classification of inflammatory diseases in general.

**Figure 2:**
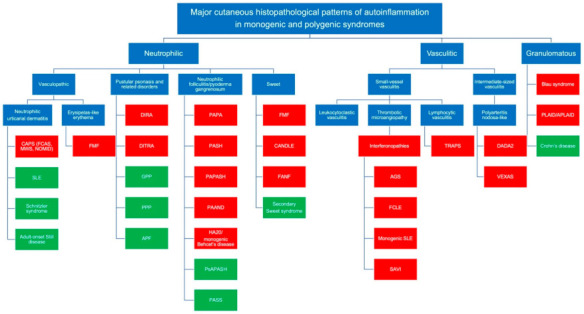
Major cutaneous histopathological patterns (neutrophilic, vasculitic and granulomatous) seen in autoinflammation in both monogenic autoinflammatory syndromes (Red) and complex multigenic diseases (Green); AGS, Aicardi Goutières syndrome; APF, Amicrobial pustulosis of the folds; APLAID, Autoinflammation and phospholipase C gamma 2 associated antibody deficiency and immune dysregulation; CANDLE, Chronic atypical neutrophilic dermatosis with lipodystrophy and elevated temperature; CAPS, Cryopyrin-associated periodic syndromes; DADA2, Deficiency of adenosine deaminase 2; DIRA, Deficiency of IL-1 receptor antagonist syndrome; DITRA, Deficiency of IL-36 receptor antagonist; FCAS, Familial cold autoinflammatory syndrome; FCLE, Familial chilblain lupus erythematosus; FANF, Familial autoinflammatory necrotising fasciitis; FMF, Familial Mediterranean fever syndrome; GPP, Generalized pustular psoriasis; HA20, Haploinsufficiency of A20; MWS, Muckle–Wells syndrome; NOMID, Neonatal-onset multisystem inflammatory disease; PAAND, Pyrin-associated autoinflammation with neutrophilic dermatosis; PAPA, Pyogenic arthritis, pyoderma gangrenosum and acne; PAPASH, Pyogenic Arthritis, pyoderma gangrenosum, acne, suppurative hidradenitis; PASH, Pyoderma gangrenosum, acne, suppurative Hidradenitis; PASS, Pyoderma gangrenosum, acne, suppurative hidradenitis and ankylosing spondylitis; PLAID, Phospholipase C gamma 2 associated antibody deficiency and immune dysregulation; PPP, Palmoplantar pustulosis; PsAPASH, Psoriatic arthritis, pyoderma gangrenosum, acne, suppurative hidradenitis; SAVI, Stimulator of interferon genes (STING) associated vasculopathy of infancy; SLE, Systemic lupus erythematosus; TRAPS, TNF receptor-associated periodic syndrome; VEXAS, vacuoles, E1 enzyme, X-linked, autoinflammatory, somatic syndrome.

This review will focus on the identification of the three main histopathological patterns, followed by the identification of more subtle histopathological clues. These patterns will then be correlated with clinical findings, which are usually from pediatric patients. The three main histopathologic groups are: (1) neutrophilic; (2) vasculitic; and (3) granulomatous. Identification of any pattern may lead to a diagnosis of either a MAIS or a complex multigenic disease. Figure 2 delineates the histopathological patterns of autoinflammation in both major MAIS and complex multigenic diseases and Table 1 classifies the main monogenic autoinflammatory syndromes based on their mechanism of pathogenesis and relevant histopathological findings.

## 2. Autoinflammatory Diseases: Correlating Histologic Patterns with Specific Diseases

### 2.1. The Neutrophilic Pattern

The neutrophilic pattern can be further subdivided into 4 groups as follows: (a) vasculopathic; (b) pustular psoriasis; (c) aseptic neutrophilic folliculitis, including pyoderma gangrenosum (PG); and (d) Sweet’s syndrome.

#### 2.1.1. The Vasculopathic Pattern

The vasculopathic reaction pattern includes a group of diseases in which inflammation produces erythematous and slightly infiltrated plaques, resulting from vasodilatation, increased vessel wall permeability with resulting slight dermal edema. In contrast to the ‘vasculitic’ pattern, there is no vessel wall damage and the lesions resolve rapidly. The vasculopathic pattern contains the spectrum of diseases in neutrophilic urticarial dermatitis (NUD), and the erysipelas-like erythema of the familial Mediterranean fever (FMF) syndrome. The NUD group contains the spectrum of cryopyrin-associated periodic syndromes (CAPS) or ‘cryopyrinopathies’. CAPS includes three main diseases: (1) familial cold autoinflammatory syndrome (FCAS); (2) Muckle–Wells syndrome (MWS); and (3) neonatal onset multisystem inflammatory disorder (NOMID). The remaining diseases associated with NUD are systemic LE, Schnitzler syndrome and adult-onset Still-disease in adults. The two latter diagnoses are only seen in adults and will only be briefly mentioned.

NUD is characterized by chronic, recurrent urticarial wheals lasting 24–48 h. Unlike usual urticaria, the lesions may last more than 24 h and are not responsive to antihistamine treatment. Of note, the eruption has a diurnal variation, being absent or discrete in the morning but gradually increasing in severity into the evenings. Histopathological examination of NUD shows no epidermal change or subjacent edema. There is a dense, perivascular and interstitial neutrophilic infiltrate with leukocytoclasia. The neutrophils may show a linear interstitial arrangement between collagen bundles. Neutrophils may show epitheliotropism and a peri-eccrine accentuation (‘neutrophilic hidradenitis’) [11,12]. Dilated vessels often contain neutrophils. No true leukocytoclastic vasculitis is present with fibrin in venule walls. A few eosinophils may be present [13,14].

**Figure 3:**
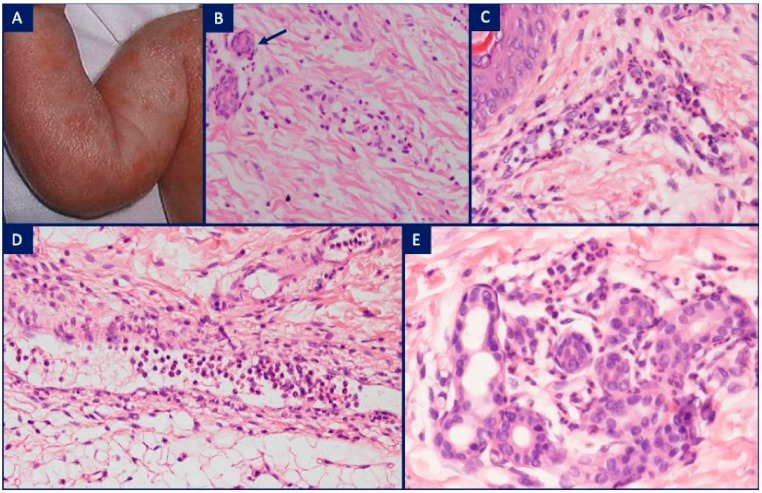
Neutrophilic urticarial dermatitis (NUD) in neonatal-onset multisystem inflammatory disease (NOMID). (**A**) Female newborn with an urticarial eruption associated with recurrent fever, increased serologic inflammatory markers, and aseptic purulent meningitis; (**B**) H&E, ×10. A perivascular, interstitial and peri-eccrine (arrow) mononuclear cell infiltrate admixed with neutrophils and rare eosinophils; (**C**) H&E, ×20. A perivascular neutrophilic infiltrate with leukocytoclasia but no fibrinoid necrosis in vessel walls; (**D**) H&E, ×10. Dilated lymphatics containing numerous neutrophils; (**E**) H&E, ×20. Peri-eccrine neutrophilic hidradenitis.

CAPS are due to mutations leading to cryopyrin activation. There exists a spectrum of different disease-severity syndromes, from mild disease in the FCAS; intermediate disease in MWS; and severe disease in NOMID [15,16]. FCAS starts in the first year of life with an urticarial eruption and flu-like symptoms induced by cold stimuli, including ingestion of cold liquid [17]. A less common variant, termed ‘FCAS2′ (NLRP12-related disease), has a later onset in childhood with symptoms lasting 2–10 days [18]. MWS is intermediate in terms of disease severity and characterized by fever, a chronic evanescent urticarial eruption, arthralgia, conjunctivitis and systemic AA amyloidosis, often leading to renal impairment. Fever may be absent. Symptoms usually appear within the first 6 months of life but may also develop later in adolescence. In contrast to FCAS, attacks in MWS are not cold-induced, and the episodes tend also to last longer, usually 1 to 2 days. Similar to FCAS, patients with MWS describe a pattern of worsening symptoms in the evening. The course of the disease varies among individuals, ranging from recurrent attacks to near-continuous symptoms [16]. NOMID, is also called chronic infantile neurologic cutaneous and articular syndrome (CINCA). NOMID is the most severe form of CAPS and is characterized by an urticarial eruption associated with arthralgia and neurologic manifestations. The onset is within 6 months of age with two thirds of patients being affected at birth, or even in utero. The duration of attacks is usually less than 24 h. The initial presentation is similar to other CAPS disease with variable degrees of articular and neurologic involvement [19,20]. A histologic finding of NUD with a peri-eccrine neutrophilic hidradenitis in the correct clinical setting should raise suspicion of NOMID (Figure 3) [12,21].

Non-bullous neutrophilic LE, a variant of systemic LE which clinically and histopathologically resembles NUD, is a cutaneous marker suggesting progression to systemic disease. On histology, there is a vacuolar interface dermatitis with apoptotic keratinocytes in addition to typical NUD. Direct immunofluorescence studies show granular IgG deposition along the dermal-epidermal junction. Neutrophil-rich lupus panniculitis can also be seen in the context of progression to systemic LE, in contrast to the usual lymphocytic panniculitis seen in chronic cutaneous LE [22].

NUD is also seen in Schnitzler syndrome and adult-onset disease, which are only briefly mentioned because they appear in adulthood. Schnitzler syndrome is characterized by chronic urticaria associated with recurrent fever, lymphadenopathy, increased erythrocyte sedimentation rate, leukocytosis and a monoclonal gammopathy, which is usually IgM, or rarely, IgG in the ‘Schnitzler variant’. The disease may progress to a blood dyscrasia, especially ‘Waldenstrom’ macroglobulinemia. Histopathology in Schnitzler syndrome shows typical NUD or mononuclear-cell infiltrate with a perivascular accentuation. Eosinophils are sparse or absent [23]. Adult-onset Still’s disease has a ‘typical’ and an ‘atypical’ clinical presentation. The typical presentation has an evanescent urticarial eruption of NUD. The atypical presentation has more persistent lesions, often in a photo-distributed pattern with a reticular or rippled-like pattern and associated with a more aggressive disease course and associated malignancy (lymphoma, breast cancer). In addition to histologic features of NUD, the ‘atypical’ eruption also shows psoriasiform hyperplasia with focal parakeratosis, dyskeratotic keratinocytes in the superficial epidermis, and small intraepidermal clusters of neutrophils. The dermis has superficial perivascular lymphocytes with or without interstitial neutrophils [24].

**Figure 4:**
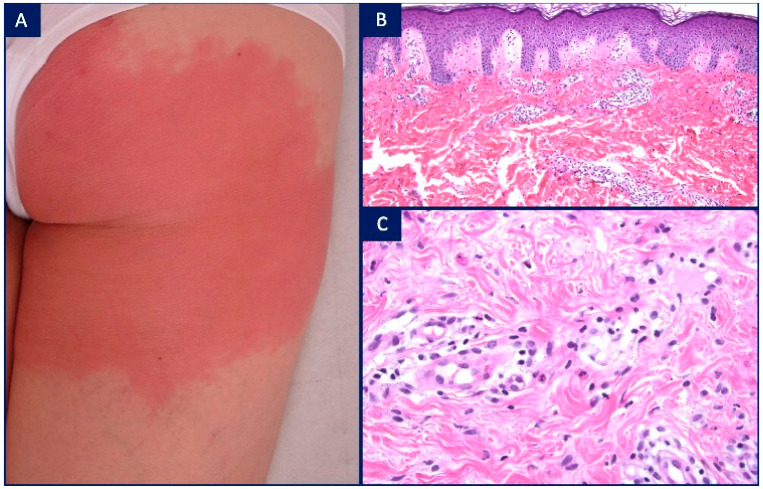
Erysipelas-like erythema in familial Mediterranean fever syndrome (FMF): (**A**) 34-year-old female with a recurrent, well-demarcated erythematous plaque on the right buttock, spontaneously resolving within 2–3 days; (**B**) H&E, ×4. Mild papillary dermal edema, dilated blood and lymphatic vessels surrounded by a mild inflammatory cell infiltrate; (**C**) H&E, ×20. Perivascular infiltrate of lymphocytes and neutrophils with slightly thickened vessel walls, but devoid of fibrin deposition.

The final disease in the vasculopathic group is erysipelas-like erythema (ELE), which is a minor diagnostic criterion for the diagnosis of the familial Mediterranean fever (FMF) syndrome. Clinically, FMF has recurrent, self-limited attacks of fever for ~3 days with polyserositis involving the peritoneum, pleura and synovium [25,26,27]. There is a favorable response to colchicine and progression to amyloidosis if untreated. ELE is the only pathognomonic cutaneous marker of FMF and is seen in a minority of cases of FMF (7–46% of all patients and 15–20% of children). ELE typically occurs on the lower extremities with well-demarcated plaques, no larger than 15 cm in diameter, and it may be triggered by physical exercise. The lesions subside with rest over 48–72 h [28]. Histopathological findings for ELE are subtle with no epidermal change, mild papillary dermal edema, dilated blood and lymphatic vessels and a perivascular infiltrate of lymphocytes and neutrophils with variable amounts of leukocytoclasia. Eosinophils are absent (Figure 4) [29]. Endothelial cells may appear swollen. Although blurring of some vessel walls may be observed, no fibrin deposition occurs and leukocytoclastic vasculitis is not observed. This may be due to the short duration of the attacks, which is insufficient to provoke vessel wall damage [29,30]. However, direct immunofluorescence shows deposits of IgM, C3, and fibrinogen in the small-sized vessel walls within the papillary dermis [28,30]. A recurrent subepidermal bullous eruption has been reported with ELE with negative direct immunofluorescence study findings [31].

#### 2.1.2. The Pustular Psoriasis Pattern

Pustular psoriasis with its related disorders is the second type of neutrophilic pattern. This pattern occurs in the deficiency of IL-1 receptor antagonist syndrome (DIRA), deficiency of IL-36 receptor antagonist syndrome (DITRA), pustular psoriasis (including generalized pustular psoriasis and palmoplantar pustulosis), and amicrobial pustulosis of the skin folds.

DIRA is a recessive loss-of-function mutation of the IL-1RN gene, which encodes the IL-1 receptor antagonist. The result is neonatal generalized pustular psoriasis, fever with elevated inflammatory markers, joint swelling, multifocal osteolytic lesions, hepatosplenomegaly and interstitial lung disease. The outcome is poor, leading to death with multi-organ failure. The rash mostly involves the face including oral and conjunctival involvement, and there is also diffuse pustular psoriasis with erythematous, scaly plaques. Nails may be involved. Pathergy may also be present. Histopathologically, the changes are identical to pustular psoriasis with psoriasiform epidermal hyperplasia, neutrophils in parakeratotic foci, and spongiform subcorneal pustules. In contrast to usual pustular psoriasis, neutrophils may densely infiltrate the dermis and show peri-eccrine accentuation. Leukocytoclastic vasculitis has also been reported, with vasculitis being present even in the deeper subcutaneous adipose tissue adjacent to bone [32,33].

**Figure 5:**
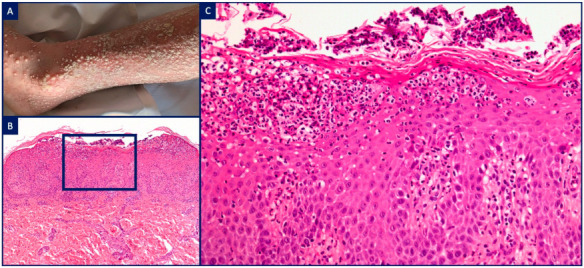
Pustular-psoriasis in deficiency of IL-36 receptor antagonist syndrome (DITRA): (**A**) 5-year-old boy with an erythrodermic eruption showing confluent, non-follicular, aseptic pustules on an erythematous background; (**B**) H&E, ×4. Psoriasiform epidermal hyperplasia with aggregates of subcorneal and intra-epidermal neutrophils; (**C**) H&E, ×10. Spongiform pustules, neutrophils in a parakeratotic stratum corneum, and a neutrophilic infiltrate in the papillary dermis. Courtesy of Deborah Salik.

IL-36RN is a negative regulator of IL-36 receptor signaling, and loss of function is present in the monogenic syndrome called DITRA and in complex multigenic diseases such as generalized pustular psoriasis, palmoplantar pustular psoriasis, and acrodermatitis continua of Hallopeau. DITRA is characterized by recurrent episodes of generalized pustular psoriasis, fever, systemic inflammation and leukocytosis. Histopathological findings, like DIRA, are identical to pustular psoriasis though the density of the neutrophilic infiltrate in the papillary dermis appears to be denser than in pustular psoriasis (Figure 5) [34,35].

CARD14 gain-of-function mutations result in a monogenic syndrome called CARD-14-associated-papulosquamous eruption (CAPE) and the complex multigenic diseases of generalized pustular psoriasis and palmoplantar pustular psoriasis [35,36,37]. CAPE is identical to pityriasis rubra pilaris (PRP) type V, which causes a hereditary, pediatric form of PRP. There is early onset in life with prominent facial involvement, typically sparing the lower lip, and associated arthritis. The patients have features of psoriasis, PRP, or a combination of both with erythroderma [38,39]. The histopathological findings for CAPE are identical to PRP, showing psoriasiform hyperplasia with irregular hyperkeratosis, alternating vertical and horizontal orthokeratosis and parakeratosis and follicular plugging. In contrast to typical PRP, both psoriasis and CAPE lack acantholysis [40].

Amicrobial pustulosis of the folds and the scalp, rather than occurring in monogenic diseases, occurs in a few complex multigenic inflammatory diseases, most frequently systemic LE, mixed connective tissue disease, Sjögren syndrome and Crohn’s disease. Age ranges from 12 to 63-years-old (mean 30 years). Clinically, there are non-follicular-based pustules on an erythematous ground. Histopathology shows spongiform pustulation identical to pustular psoriasis [41].

#### 2.1.3. The Aseptic Neutrophilic Folliculitis Pattern

Aseptic neutrophilic folliculitis is the third type of neutrophilic pattern. Aseptic neutrophilic folliculitis is the primary lesion seen in PG, hidradenitis suppurativa (HS) and acne.

The folliculitis may occur in a variety of overlapping syndromes that show neutrophilic folliculitis, pyogenic arthritis and seronegative spondyloarthropathies including ankylosing spondylitis and psoriatic arthritis. The syndromes have been grouped in a set of so-called ‘P’ syndromes as follows: PAPASH (pyogenic arthritis, pyoderma gangrenosum, acne, suppurative hidradenitis); PsAPASH (psoriatic arthritis, pyoderma gangrenosum, acne, suppurative hidradenitis); PASS (pyoderma gangrenosum, acne, suppurative hidradenitis and ankylosing spondylitis); PASH (pyoderma gangrenosum, acne, suppurative hidradenitis); and PAPA (pyogenic arthritis, pyoderma gangrenosum, acne) [42]. PAPASH, PASH and PAPA syndromes are all MAIS [43,44]. PAPA syndrome is variably expressed with onset of joint disease between 1 and 16 years of age and acne beginning in puberty, associated with mild physical trauma. Severe, cystic, disfiguring acne appears after the joint disease [45,46].

Some consider erosive pustular dermatosis of the scalp to be a neutrophilic folliculitis similar to PG, especially because it may start after mechanical trauma (younger patients) or treatment of actinic keratoses (older patients). It is possible, however, that the disease in younger patients is an aseptic neutrophilic folliculitis, while the disease in older patients is the result of an inability to maintain epithelialization in severely actinic-damaged skin. Reports of histopathologic findings, though, do propose that the primary lesion may be, similarly to PG, a neutrophilic folliculitis [47].

Pyrin-associated auto-inflammation with neutrophilic dermatosis (PAAND) is due to mutations in the MEFV gene. Features of FMF such as amyloidosis and serositis are absent. PAAND is characterized by childhood-onset of recurrent episodes of PG, acne, fever (which last for weeks as opposed to days in FMF), elevated acute-phase reactants, arthralgia, and myalgia/myositis and pyogenic arthritis. Leukocytoclastic vasculitis, reported as ‘neutrophilic small-vessel vasculitis’, has also been described (see below) [48].

Haploinsufficiency of A20 (HA20) causes recurrent oral and genital ulcers mimicking the bipolar aphthosis of Behçet’s disease. Although the histopathological findings for this syndrome have not yet been elucidated, this syndrome can be included in this group since mucosal ulceration, acne and folliculitis have been described [49]. Monogenic Behçet’s disease can be also caused by rare, identified mutations in genes of the NF-κB pathway [50].

#### 2.1.4. Sweet’s Syndrome

Sweet’s syndrome is the last group of neutrophilic patterns. Various MAIS produce the neutrophilic, histiocytoid and necrotizing variants. FMF can produce the neutrophilic variant [51]. Chronic atypical neutrophilic dermatosis with lipodystrophy and elevated temperature (CANDLE) syndrome is similar to the histiocytoid variant of Sweet’s syndrome. CANDLE causes annular violaceous plaques, persistent eyelid swelling and lipodystrophy. Histopathological findings for CANDLE show a superficial and deep dermal interstitial infiltrate of CD68+, CD163+, myeloperoxidase+, CD45+ mononuclear myeloid cells, admixed with leukocytoclasia [52]. Necrotizing Sweet syndrome, often misdiagnosed as infectious necrotizing fasciitis, is a diagnostic pitfall, and occurrence after a surgical procedure or a trauma is an important clinical clue. A careful clinical correlation can help exclude a hematologic disorder, particularly myelodysplastic syndrome, a connective tissue disease, an endocrine disorder, or a drug eruption (granulocyte colony-stimulating factor) [53]. Familial autoinflammatory necrotizing fasciitis is a recently described NFkBopathy [50,54]. Recurrent sterile necrotizing fasciitis should lead one to suspect this disease, since an aggressive surgical therapeutic approach after a misdiagnosis of a bacterial infection can cause rapid disease progression and death.

### 2.2. The Vasculitic Pattern

The second histopathological pattern leading to suspicion of autoinflammation is vasculitis and it can be seen in either small-sized vessels (capillaries and post-capillary venules), or intermediate-sized vessels that have muscular walls (arterioles).

#### 2.2.1. Small Sized-Vessel Vasculitis

The MAIS that produce small-sized vessel vasculitis include leukocytoclastic vasculitis, the thrombotic microangiopathy seen in the interferonopathies, and the lymphocytic vasculitis seen in tumor necrosis factor receptor-associated periodic syndrome (TRAPS).

Leukocytoclastic vasculitis is a non-specific cutaneous marker of auto-inflammation and has been described in various MAIS, including FMF, PAAND, NOMID, PAPA and hyperimmunoglobulinemia D syndrome (HIDS) [55]. HIDS, also called mevalonate kinase deficiency, is characterized by recurrent fever lasting from 5 to 7 days, cervical lymphadenopathy and splenomegaly, joint disease, and gastrointestinal symptoms. Cutaneous lesions have been reported as morbilliform, urticarial and purpuric. More frequently, however, the lesions lack a morphological description and are reported simply as a ‘rash’ [56,57,58]. Histopathology shows small vessel vasculitis, including Henoch–Schonlein purpura and erythema elevatum diutinum [58,59,60].

**Figure 6:**
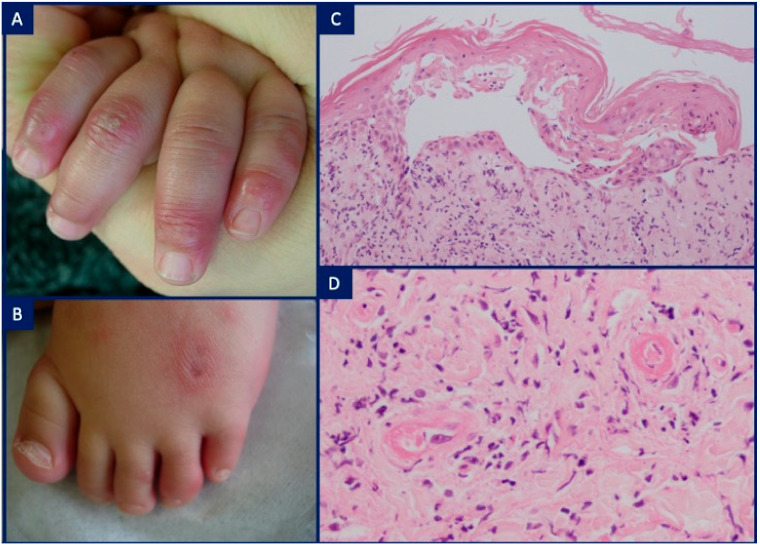
Thrombotic microangiopathy in Aicardi–Goutières syndrome: (**A**,**B**) 1-year-old female with acral necrotic bullous chilblains (**C**) H&E, ×4. Vacuolar interface dermatitis associated with numerous apoptotic keratinocytes, epidermal necrosis and subepidermal bulla. similar to necrotic chilblain lupus erythematosus; (**D**) H&E, ×20. Fibrin deposition in small-sized vessel walls associated with intraluminal thrombi.

Thrombotic microangiopathy is the histopathological hallmark of the interferonopathies, all characterized by mutations that lead to the aberrant release of type I IFN and inflammation simulating viral infection or systemic LE. The thrombotic microangiopathy in the interferonopathies causes severe necrotic chilblains, leading to digital amputations and ear-tissue loss. The patients also present with features of systemic lupus erythematosus (i.e., antinuclear antibodies specific for SLE) [61]. Monogenic systemic LE with cytopenia, glomerulonephritis, arthritis and oral ulcers also belongs to this group of MAIS [1,62]. Aicardi–Goutières syndrome and familial chilblain lupus are allelic phenotypes of the same disease [63,64]. Patients with Aicardi–Goutières syndrome also develop severe neurological disease with intracranial basal ganglia calcifications [65,66]. Stimulator of interferon genes (STING)-associated vasculopathy with onset in infancy (SAVI) has interstitial lung disease and may be misdiagnosed as granulomatosis with polyangiitis [67,68,69,70]. Finally, spondyloenchondrodysplasia is characterized by skeletal dysplasia, and neurological developmental delay with intracranial calcification [61]. Histopathological findings for Aicardi–Goutières syndrome and familial chilblain lupus are identical to chilblain LE. There is vacuolar interface dermatitis, absent or mild papillary dermal edema and a superficial and deep lymphocytic infiltrate, often with a peri-eccrine accentuation. There may also be a lymphocytic vasculitis, with thick vessel walls, red cell extravasation and formation of intraluminal thrombi (Figure 6) [61,65].

TRAPS, another of the MAIS, is characterized by prolonged episodes of fever and systemic inflammation (myalgia, arthralgia, fasciitis, pericarditis, periorbital edema and conjunctivitis). Abdominal pain is the most prominent finding. Eighty percent of cases occur in childhood, with the episodes lasting 2–4 weeks, with intervals from months to years. Periorbital edema and cellulitis-like subcutaneous inflammation of the upper limbs is a characteristic feature. The cutaneous lesions start as erythematous macules and patches, which are variable in size. They may migrate proximal-to-distal on the extremity [71,72,73]. Histopathology shows a superficial and deep lymphocytic infiltrate with tight venule cuffing but no fibrin within vessel walls [73,74].

#### 2.2.2. Intermediate-Sized Vessel Vasculitis

The MAIS that cause intermediate-sized vessel vasculitis are deficiency in adenosine deaminase 2 (DADA2) and VEXAS (vacuoles, E1 enzyme, X-linked, autoinflammatory, somatic) syndrome. Histopathology shows features identical to cutaneous polyarteritis nodosa (PAN).

**Figure 7:**
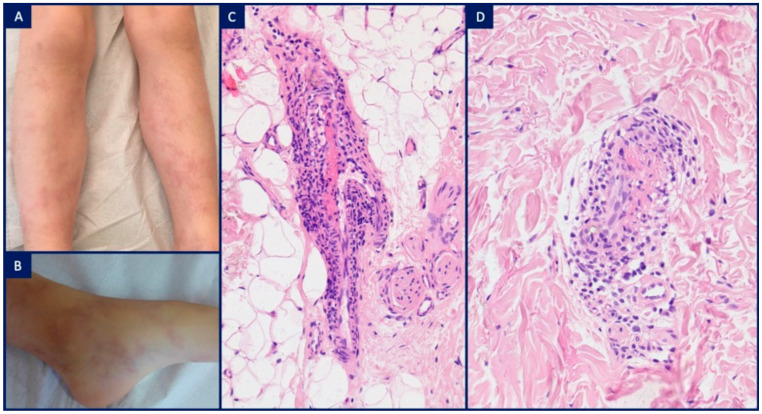
Deficiency of adenosine deaminase 2 syndrome (DADA2): (**A**,**B**) 6-year-old boy with livedo racemosa on the lower limbs; (**C**,**D**) H&E, ×10 (**C**), ×20 (**D**). Intraluminal thrombi within intermediate-sized vessels at the dermal-subcutaneous junction and a surrounding mononuclear cell infiltrate simulating lymphocytic thrombophilic arteritis.

DADA2 or monogenic polyarteritis is an autosomal recessive vasculopathy due to CECR1 (cat eye syndrome chromosome region, candidate 1) mutation. The disease is characterized by PAN, lymphopenia and pancytopenia, and a B-cell immunodeficiency [75,76]. The vasculitis starts early in life and is characterized by the triad of livedo racemosa, recurrent fever and strokes [77,78,79]. DADA2 also has microangiopathic involvement, with clinical findings similar to the interferonopathies with necrotic chilblains [77,80]. Importantly, any diagnosis of PAN in a child should always exclude DADA2, since early implementation of anti-TNF therapy may prevent a stroke. Histopathological findings for DADA2 show a neutrophilic infiltrate with the neutrophils being present in and around and muscular arteriole walls where there is fibrinoid necrosis. The internal elastic lamina is disrupted, and there are hyalin fibrin rings with intraluminal thrombi [79,81]. We have also been confronted by a case of DADA2 in a child with extensive livedo, whose skin biopsy revealed a mononuclear cell infiltrate surrounding thrombosed intermediate-sized vessels simulating lymphocytic thrombophilic arteritis (Figure 7).

The VEXAS syndrome is caused by mutations of the UBA1 gene. No pediatric cases have been reported. VEXAS syndrome is characterized by fever, cytopenias, characteristic vacuoles in myeloid and erythroid precursor cells, dysplastic bone marrow (myelodysplastic syndrome or multiple myeloma), relapsing polychondritis, Sweet’s syndrome, polyarteritis nodosa, and giant-cell arteritis [82].

### 2.3. The Granulomatous Pattern

The granulomatous pattern is the third pattern seen in autoinflammation. This pattern is seen with NOD2 mutations in Blau syndrome and with PLCƔ2 mutations in ‘autoinflammation and phospholipase C gamma 2 associated antibody deficiency and immune dysregulation’ (APLAID) and ‘phospholipase C gamma 2 associated antibody deficiency and immune dysregulation’ (PLAID). Both NOD2 and PLCƔ2 mutations are associated with Crohn’s disease [83,84].

Blau syndrome (early-onset sarcoidosis) is characterized by the triad of articular, cutaneous and ocular non-caseating granulomatous inflammation. The disease begins very early in life. The cutaneous lesions initially are monomorphic, non-confluent papules, and late lesions are heterogenous with confluent papules, plaques, nodules and panniculitis. Histopathological findings are consistently sarcoidal granulomas in both early and late lesions [85,86,87]. Granulomas in Blau syndrome and granulomas in NOD2-associated Crohn’s disease show distinct morphologic features with granulomas with large lymphocytic coronas, emperipolesis of lymphocytes within multinucleated giant cells and giant cell apoptosis seen only in Blau syndrome [88]. In contrast to Blau syndrome, NOD2-associated autoinflammatory syndrome (NAID) occurs in adults. NAID has gastro-intestinal symptoms instead of uveitis with cutaneous erythematous patches and plaques. Histopathological findings in NAID show a non-specific spongiotic dermatitis [89]. Patients may show phenotypical overlap between Blau syndrome, NAID, and Crohn’s disease [84,86,90,91].

PLAID and APLAID are allelic diseases that are both caused by PLCƔ2 mutations, which produce a B-cell immunodeficiency. Mutations responsible for PLAID are deletions that result in enhanced PLCƔ2 signaling and mutations responsible for APLAID are missense mutations that decrease the threshold required for PLCƔ2 activation. PLAID is associated with autoimmune diseases (thyroiditis, vitiligo, positive antinuclear antibodies) and cold-induced urticaria. The histopathological findings for cold-induced urticaria are similar to the NUD already discussed in the cryopyrinopathy section. Neonates with PLAID also have blistering ulcerations, especially on the nose, ears and fingers. Ulceration usually spontaneously resolves, but, in a few cases, it may progress, thereby leading to destruction of ear and nose cartilage. In neonates with self-resolving ulceration, isolated granulomatous patches may develop later in life. The histopathology of these patches shows non-caseating granulomatous inflammation. APLAID is associated with a neonatal-onset vesicular-bullous eruption and recurrent acral hemorrhagic blisters. Histopathology in APLAID shows a dense perivascular and interstitial granulomatous infiltrate, palisading granulomas around areas of collagen necrobiosis, and a dense neutrophilic infiltrate. Leukocytoclastic vasculitis is also observed. Bulla formation can result from intense papillary dermal edema [92,93,94,95].

## 3. Conclusions

Autoinflammation can be suspected upon cutaneous histopathological examination and based on recognition of three main histopathological patterns: the neutrophilic, the vasculitic and the granulomatous patterns. In this review we have described the journey of the dermatopathologist, beginning with the identification of these histopathological patterns, followed by the identification of additional histopathological clues and the correlation of these findings with the clinical manifestations in the pediatric patient. This would lead to the identification of an activated regulatory pathway and the diagnosis of a MAIS or a complex multigenic disease.

## Figures and Tables

**Table 1:** Main histopathological findings for the major monogenic autoinflammatory syndromes with their mechanism of pathogenesis and identified mutated genes (in parenthesis): AGS, Aicardi Goutières Syndrome; APLAID, autoinflammation and phospholipase C gamma 2 associated antibody deficiency and immune dysregulation; CANDLE, Chronic atypical neutrophilic dermatosis with lipodystrophy and elevated temperature; CAPE, caspase-activating recruitment domain member 14 associated papulosquamous eruption, DADA2, deficiency of adenosine deaminase 2; DIRA, Deficiency of IL-1 receptor antagonist syndrome; DITRA, Deficiency of IL-36 receptor antagonist; FCAS, Familial cold autoinflammatory syndrome; FCLE, Familial chilblain lupus erythematosus; FMF, Familial Mediterranean fever syndrome; HA20, Haploinsufficiency of A20; MWS, Muckle–Wells syndrome; NAID, NOD-2 associated autoinflammatory syndrome; NOMID, Neonatal-onset multisystem inflammatory disease; PAAND, pyrin-associated autoinflammation with neutrophilic dermatosis; PAPA, Pyogenic Arthritis, pyoderma gangrenosum and acne; PLAID, Phospholipase C gamma 2 associated antibody deficiency and immune dysregulation; SAVI, Stimulator of interferon genes (STING) associated vasculopathy of infancy; TRAPS, TNF receptor-associated periodic syndrome.

Interleukin-1 activation	Inflammasomopathies	Pyrin activation	FMF (MEFV)	Erysipelas-like erythema in the only pathognomic cutaneous marker of FMF and is characterized by mild papillary dermal edema, dilated vessels, sparse perivascular mononuclear cell infiltrate admixed with neutrophils and nuclear dust
			PAAND (MEFV) and PAPA (PSTPIP1)	Neutrophilic dermatosis comprising the phenotypical spectrum of pyoderma gangrenosum, neutrophilic folliculitis, abscess and acne, PAAND also shows small vessel leukocytoclastic vasculitis
		Cryopyrin/NLRP3 activation	Cryopyrinopathies: FCAS, MWS, NOMID	Neutrophilic urticarial dermatitis: perivascular and interstitial neutrophilic infiltrate with variable leukocytoclasia, linear interstitial arrangement of neutrophils as ‘Indian file’, neutrophilic epitheliotropism including intraepidermal and peri-eccrine involvement, absence of fibrin in vessel walls, no significant dermal edema
	IL1 receptor antagonist		DIRA (ILRN)	Pustular psoriasis (psoriasiform epidermal hyperplasia, subcorneal spongiform pustulation, neutrophilic aggregates within parakeratotic stratum corneum), dense intradermal neutrophilic infiltrate with peri-eccrine involvement
	IL36 receptor antagonist		DITRA (IL36RN)	
	Phospholipase C gamma 2 activation		APLAID (PLCG2)	Dense perivascular and interstitial granulomatous infiltrate, palisading granulomas around necrobiotic collagen, dense neutrophilic infiltrate, intense papillary dermal oedema leading to subepidermal blistering
			PLAID (PLCG2)	Neutrophilic urticarial dermatitis (cold-induced urticaria) and non-caseating sarcoidal granulomas
Type I Interferon activation		Intracytoplasmic nucleic acid accumulation	AGS (TREX1, RNASEH2A, RNASEH2B, RNASEH2C, SAMHD1, ADAR1, IFIH1) and FCLE (TREX1, SAMHD1)	Thrombotic microangiopathy associated with findings of chilblain lupus erythematosus (vacuolar interface dermatitis, lymphocytic vasculitis, peri-eccrine lymphocytic infiltrate)
		Increased intracytoplasmic nucleic acid sensing	SAVI (TMEM137)	Thrombotic microangiopathy
		Proteasome dysfunction	CANDLE (PSMB8)	Histiocytoid Sweet syndrome: dense perivascular and interstitial mononuclear cell infiltrate composed of immature myeloid cells admixed with mature neutrophils, eosinophils and leukocytoclasia
NF-κB activation		TNF receptor 1 activation	TRAPS (TNFRSF1A)	Lymphocytic vasculitis: perivascular lymphocytic infiltrate showing tight cuffing within both superficial and deep dermis, absence of fibrin within vessel walls
		NOD2 activation	Blau (NOD2)	Non-caseating sarcoidal granulomas with lymphocytic coronas
			NAID (NOD2)	Subacute spongiotic dermatitis, irregular epidermal acanthosis with overlying parakeratotic hyperkeratosis
		CARD14 activation	CAPE (CARD14)	Pityriasis rubra pilaris (psoriasiform epidermal hyperplasia, irregular hyperkeratosis, alternating vertical and horizontal orthokeratosis and parakeratosis, follicular plugging), absence of acantholysis
		A20 insufficiency	HA20 (TNFAIP3)	Non-specific (oral aphtous erosions or ulcerations similarly to Behcet’s disease)
M1 macrophage activation		Deficiency adenosine deaminase 2	DADA2 (CECR1)	Polyarteritis nodosa: neutrophilic infiltrate around and within muscular arteriole walls (intermediate-sized vessels at the dermo-hypodermal junction), disruption of the internal elastic lamina, fibrin deposition and intraluminal thrombosis

## Abbreviations

APLAID	autoinflammation and phospholipase C gamma 2 associated antibody deficiency and immune dysregulation
CANDLE	Chronic Atypical Neutrophilic Dermatosis with Lipodystrophy and Elevated temperature
CAPE	caspase-activating recruitment domain, member 14 associated papulosquamous eruption
CAPS	cryopyrin-associated periodic syndromes
DADA2	deficiency of adenosine deaminase 2
DIRA	Deficiency of IL-1 Receptor Antagonist syndrome
DITRA	Deficiency of IL-36 Receptor Antagonist
FCAS	Familial Cold Autoinflammatory syndrome
FMF	Familial Mediterranean Fever syndrome
HA20	Haploinsufficiency of A20
HIDS	Hyperimmunoglobulinemia D syndrome
IFN	interferon
IL	interleukin
LE	lupus erythematosus
MAIS	monogenic autoinflammatory syndromes
MWS	Muckle–Wells syndrome
NOMID	neonatal-onset multisystem inflammatory disease
PAAND	pyrin-associated autoinflammation with neutrophilic dermatosis
PAPA	Pyogenic Arthritis, Pyoderma gangrenosum and Acne
PAPASH	Pyogenic Arthritis, Pyoderma gangrenosum, Acne, Suppurative Hidradenitis
PASH	Pyoderma gangrenosum, Acne, Suppurative Hidradenitis
PASS	Pyoderma gangrenosum, Acne, Suppurative hidradenitis and ankylosing Spondylitis
PLAID	Phospholipase C gamma 2 associated antibody deficiency and immune dysregulation
PLCƔ2	Phospholipase C gamma 2
PsAPASH	Psoriatic Arthritis, Pyoderma gangrenosum, Acne, Suppurative Hidradenitis
SAVI	Stimulator of interferon genes (STING) associated vasculopathy of infancy
TRAPS	TNF receptor-associated periodic syndrome
VEXAS	Vacuoles, E1 enzyme, X-linked, Autoinflammatory, Somatic syndrome
